# The impact of intravenous iron supplementation in elderly patients undergoing major surgery

**DOI:** 10.1186/s12877-022-02983-y

**Published:** 2022-04-07

**Authors:** Lea Valeska Blum, Philipp Zierentz, Lotta Hof, Jan Andreas Kloka, Leila Messroghli, Kai Zacharowski, Patrick Meybohm, Suma Choorapoikayil

**Affiliations:** 1 Department of Anaesthesiology, Intensive Care Medicine and Pain Therapy, University Hospital Frankfurt, Goethe University Frankfurt, Theodor-Stern-Kai 7, 60590 Frankfurt, Germany; 2 Department of Anaesthesiology, Intensive Care, Emergency and Pain Medicine, University Hospital Wuerzburg, Wuerzburg, Germany

**Keywords:** Iron deficiency, Elderly, Geriatric patients, Surgery, Patient care, Intravenous iron

## Abstract

**Background:**

Age and preoperative anaemia are risk factors for poor surgical outcome and blood transfusion. The aim of this study was to examine the effect of iron supplementation in iron-deficient (ID) elderly patients undergoing major surgery.

**Method:**

In this single-centre observational study, patients ≥ 65 years undergoing major surgery were screened for anaemia and ID. Patients were assigned to the following groups: A^−^ (no anaemia); A^−^,ID^+^,T^+^ (no anaemia, iron-deficient, intravenous iron supplementation); A^+^ (anaemia); and A^+^,ID^+^,T^+^ (anaemia, iron-deficient, intravenous iron supplementation).

**Results:**

Of 4,381 patients screened at the anaemia walk-in clinic, 2,381 (54%) patients were ≥ 65 years old and 2,191 cases were included in analysis. The ID prevalence was 63% in patients with haemoglobin (Hb) < 8 g/dl, 47.2% in patients with Hb from 8.0 to 8.9 g/dl, and 44.3% in patients with Hb from 9 to 9.9 g/dl. In severely anaemic patients, an Hb increase of 0.6 (0.4; 1.2) and 1.2 (0.7; 1.6) g/dl was detected with iron supplementation 6–10 and > 10 days before surgery, respectively. Hb increased by 0 (-0.1; 0) g/dl with iron supplementation 1–5 days before surgery, 0.2 (-0.1; 0.5) g/dl with iron supplementation 6–10 days before surgery, and 0.2 (-0.2; 1.1) g/dl with supplementation > 10 days before surgery (*p* < 0.001 for 1–5 vs. 6–10 days). Overall, 58% of A^+^,ID^+^,T^+^ patients showed an Hb increase of > 0.5 g/dl. The number of transfused red blood cell units was significantly lower in patients supplemented with iron (0 (0; 3)) compared to non-treated anaemic patients (1 (0; 4)) (*p* = 0.03). Patients with iron supplementation > 6 days before surgery achieved mobility 2 days earlier than patients with iron supplementation < 6 days.

**Conclusions:**

Intravenous iron supplementation increases Hb level and thereby reduces blood transfusion rate in elderly surgical patients with ID anaemia.

**Supplementary information:**

The online version contains supplementary material available at 10.1186/s12877-022-02983-y.

## Introduction

Anaemia is a common condition in elderly patients and is often considered a consequence of aging. Clinicians often accept anaemia as an accompanying disorder without further medical consideration. However, numerous studies have repeatedly demonstrated that preoperative anaemia is one of the strongest predictors of red blood cell (RBC) transfusion and postoperative morbidity. Particularly in elderly patients, anaemia is associated with increased vulnerability to adverse outcomes [1–4]. For example, Musallam et al. analysed more than 220,000 non-cardiac surgical patients and revealed that severe anaemia led to a 12.5-fold (from 0.8 to 10%) increase in 30-day mortality rate. Strikingly, even the presence of mild anaemia resulted in a 4-fold (from 0.8 to 3.5%) increase in 30-day mortality risk [5]. Similar observations were made in a study including over 70,000 patients > 65 years of age with acute myocardial infarction. Low haematocrit values were associated with an increased rate of complications including shock, heart failure, mortality, and prolonged length of stay (LOS) [6]. Data from the Third National Health and Nutrition Examination Survey (NHANES III; >30,000 samples) revealed that 32.5% of women over 65 years of age had haemoglobin (Hb) values < 13 g/dl [7]. In addition, elderly patients exhibit various comorbidities that are associated with an increased complication rate [8], necessitating extensive care during their hospital stay. Among the most prominent challenges associated with advanced age is frailty resulting in extended recovery time from surgery [9, 10]. Forni et al. recommended a close cooperation between surgeons and geriatricians to enable a multidisciplinary approach throughout treatment. Their analysis of more than 23,500 patients revealed that postponing surgery was not associated with a higher mortality rate. In contrast, the authors found a reduced 30-day mortality rate for patients treated with a multidisciplinary approach and raised the important point that patients’ pre-existing clinical conditions may be associated with worse surgical outcomes [11]. Clinicians should therefore, employ all possible measures to identify modifiable risks during preoperative assessments.

Among elderly patients, the causes of anaemia are divided in 3 broad groups: (1) renal disease and chronic inflammation; (2) nutrient deficiency; and (3) unknown origin [12]. Anaemia of unknown origin is often multifactorial and caused by multiple morbidities, cancer, androgen or vitamin D deficiency. The most frequent nutrient deficiencies associated with anaemia include vitamin B12, folate and iron of which iron deficiency anaemia (IDA) is the most prominent [13, 14]. Oral iron supplementation is inexpensive and readily available but shows poor patient compliance due to gastrointestinal side effects limiting its efficacy [15]. In addition, long-term treatment of up to 6 months is usually required to restore iron levels adequately. Supplementation with intravenous (iv) iron in patients with iron deficiency (ID) replenishes iron stores and is associated with improved postoperative outcomes. In patients undergoing major abdominal surgery, iv iron was significantly associated with a preoperative Hb increment of 0.8 g/dl, shorter LOS (7 vs. 9.7 days) and reduced RBC transfusion (from 31.3 to 12.5%) [16]. The analysis of 447 cardiac surgical patients by Evans et al. revealed that one third of iv iron supplemented IDA patients were restored to a non-anaemic state before surgery and required fewer allogeneic blood transfusions compared to anaemic patients. Of 75 supplemented patients with a mean age of 71 (± 11) years, 68% were unresponsive to treatment. The authors hypothesized that either RBC loss was greater than patients` erythropoiesis or elevated hepcidin levels impaired erythrocyte maturation [17].

In 2014, an anaemia walk-in clinic was established at the University Hospital Frankfurt to diagnose and treat IDA patients ≥ 18 years of age undergoing major surgery [18]. We demonstrated that iv iron supplementation in these patients was associated with increased preoperative Hb and decreased RBC transfusion rate [19]. However, there is limited evidence on the prevalence of ID in elderly surgical patients and the effect of iron supplementation in these patients. We thus utilized data from the anaemia walk-in clinic to elucidate the prevalence of ID in elderly surgical patients and assess the perioperative effects of iv iron supplementation.

## Materials and methods

Data used for this retrospective analysis are part of a multicentre observational epidemiological trial focusing on the implementation of Patient Blood Management (PBM) in surgical patients (Trial registration: ClinicalTrials.gov, NCT02147795). The study protocol was approved by the ethics committee of the University Hospital Frankfurt (Ref. 318/17) and the requirement for written informed consent by patients was waived.

### Patients and procedures

Data of patients (age ≥ 65 years) scheduled for major surgery with a ≥ 10% probability RBC transfusion or ≥ 500 ml blood loss [20] and screened for preoperative anaemia and ID at the anaemia walk-in clinic [18] from November 2015 to January 2020 were included in our analysis. Details regarding the screening procedure are reported in Triphaus et al. [19]. Part of the data (November 2015 – July 2018) was recently analysed in a pilot project [19]. Briefly, iv iron supplementation resulted in a preoperative increase in median Hb levels (interquartile range (IQR)) of 0 (-0.2; 0.4) g/dl, 0 (-0.2; 0.6) g/dl, and 0.6 (-0.1; 1.3) g/dl in patients with mild (*n* = 52), moderate (*n* = 47), and severe (*n* = 4) anaemia, respectively. Furthermore, preoperative Hb level increased steadily from day 6 after iron supplementation [21]. In the present study, we focused on the effect of iv iron supplementation specifically in patients ≥ 65 years of age. Data were extracted from the electronic hospital information system.

### Classification of anaemia and iron deficiency

According to the World Health Organization (WHO) anaemia is defined as an Hb concentration < 12 g/dl in women and < 13 g/dl in men. Iron deficiency was diagnosed by laboratory results according to Munoz et al. [22] and Anker et al. [23]. Briefly, ID was defined as a serum ferritin level < 100 ng/ml and transferrin saturation < 20%. In the case of chronic kidney disease or heart failure, ID was diagnosed by a ferritin level < 300 ng/ml. In addition, a full medical history of the patient was taken into account during diagnosis. Anaemia was categorized as mild (Hb 11-11.9 g/dl for women and 11-12.9 g/dl for men), moderate (Hb 8-10.9 g/dl), and severe (Hb < 8 g/dl). Accordingly, patients were assigned to the following groups: A^−^ (no anaemia); A^−^,ID^+^,T^+^ (no anaemia, ID, and iron supplementation); A^+^ (anaemia); and A^+^,ID^+^,T^+^ (anaemia, ID, and iron supplementation). We assigned all non-treated anaemic patients irrespective of the origin of anaemia to A^+^ because a preoperative increment in Hb level or differing transfusion rate is unlikely in these patients and they are therefore comparable within the group. Other common causes of anaemia such as anaemia of inflammation, anaemia of chronic renal disease, and folate or vitamin B12 deficiency, were not primarily addressed in this study.

### Iron supplementation

Patients with ID received iv iron (ferric carboxymaltose 50 mg/ml; Vifor, Saint Galene, Switzerland) at a dose of 500 mg in 100 ml saline over 15 min or 1,000 mg in 250 ml saline over 30 min depending on the laboratory findings (Hb values, body mass index, ferritin level, transferrin saturation, transferrin receptor). Contraindications were a history of hypersensitivity reaction, acute infection with antibiotics, and iron overload or recovery disorders (e.g., hemochromatosis). During and after the iv iron supplementation, the patient was actively asked regarding any discomfort to enable immediate care in case of an adverse event. The patient`s vital signs were monitored during iv iron administration and for an additional 10 to 15 min before discharge.

### Mobility

Data from the physiotherapists’ notes were extracted from the digital medical records. Mobility was defined as walking without assistance.

### Red blood cell transfusion

RBC transfusions were performed in accordance with the previous German transfusion guidelines which recommended RBC transfusion if Hb is < 6 g/dl in asymptomatic patients, between 6 and 8 g/dl in patients with cardiovascular risk factors or clinical symptoms of anaemic hypoxia [24].

### Statistical analysis

Data were checked for normal distribution using the Shapiro-Wilk test (*p* < 0.05). Descriptive variables were analysed using means with standard deviation (SD) for normal distributed data; medians with IQRs (P25%; P75%) for non-normally distributed data, as well as counts and percentages. Statistical significance was considered when *p* < 0.05 and determined by Fisher’s exact test or Mann-Whitney U test. The Kruskal-Wallis test was applied for group comparison. For calculation of Hb increment after iv iron infusion, Hb level at day of supplementation and last Hb level (< 24 h) before surgery were used. For calculation of Hb decrease, first Hb level screened by the anaemia walk-in clinic and last Hb level (< 24 h) before surgery were used. Considering that iron-dependent maturation from erythroblasts to differentiated RBC require 4–6 days [25], we assessed the impact of iv iron supplementation on Hb, RBC transfusion and mobility in intervals of 1–5, 6–10, and > 10 days. A subgroup analysis was performed to assess transfusion rate in patients of group A^+^,ID^+^,T^−^ and A^+^,ID^+^,T^+^. Linear regression model with fixed effects was used to assess predictors for Hb increase, Hb decrease, prolonged LOS, and mobility. Patients with reoperation in the postoperative period were excluded from analysis of transfusion rate. Furthermore, patients who died or underwent reoperation during their hospital stay were excluded from the LOS and mobility analyses. All analyses and graphical illustrations were performed using R software (version 3.1–124), and Microsoft Excel (2016).

## Results

**Fig. 1:**
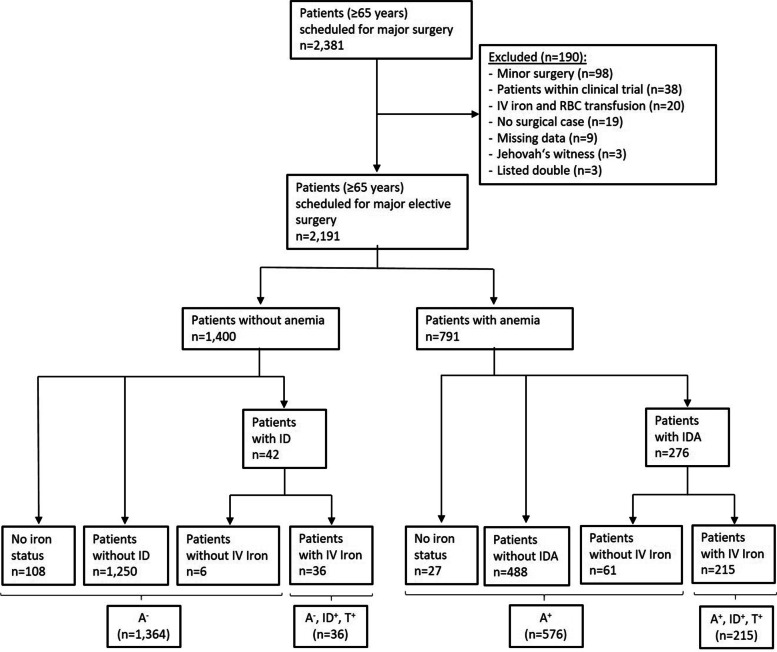
Flow chart, study population. A^−^ (no anaemia); A^−^,ID^+^,T^+^ (no anaemia, iron-deficient, iron supplementation); A^+^ (anaemia); and A^+^,ID^+^,T^+^ (anaemia, iron-deficient, iron supplementation)

**Fig. 2:**
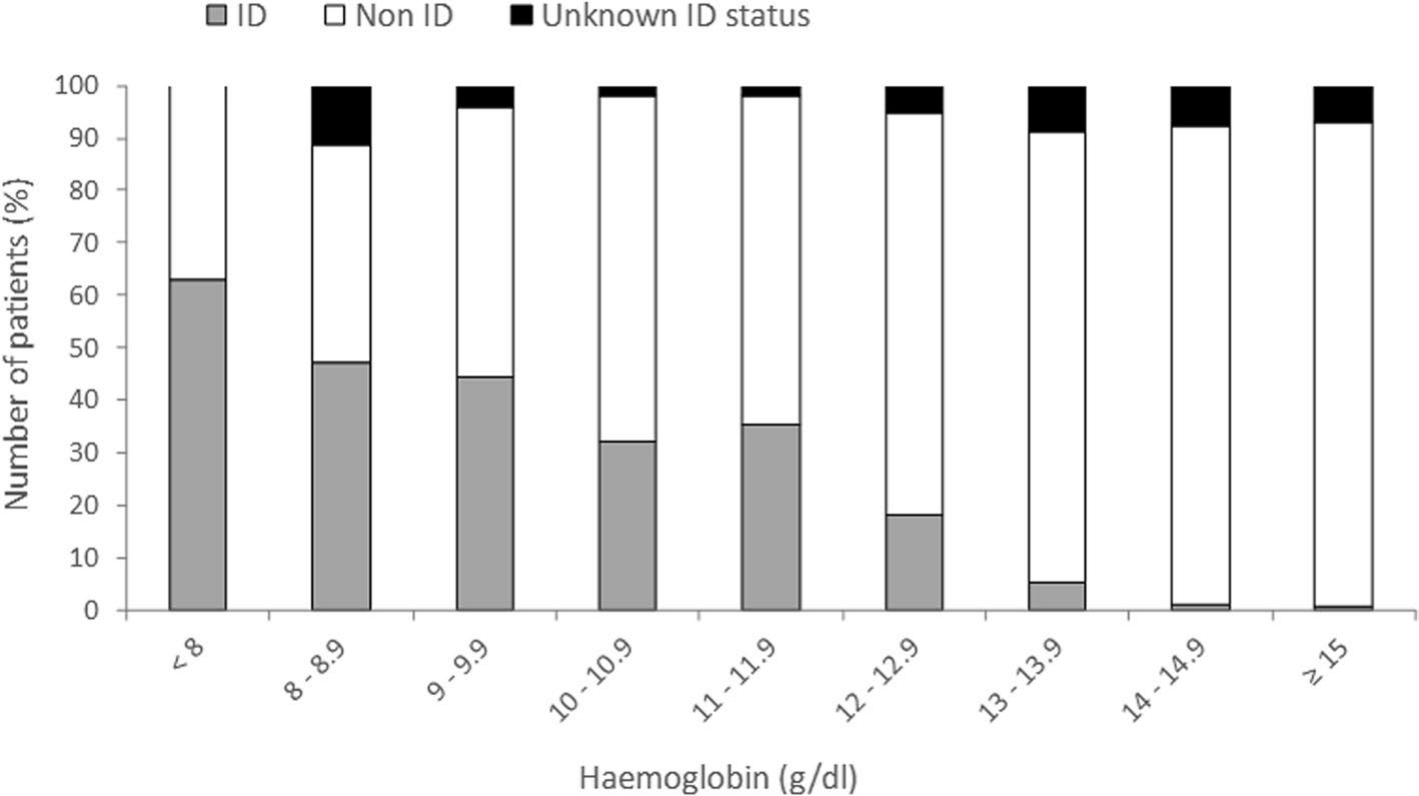
Association of iron deficiency (ID) and haemoglobin level in surgical patients (≥ 65 years)

Between November 2015 and January 2020 4,381 major surgical patients were screened for the presence of anaemia and ID, of which 2,381 patients aged ≥ 65 years were included in this study. After the exclusion of 190 patients, 2,191 cases were considered for analysis (Fig. 1). In total, 1,400 patients were non-anaemic (63.9%), of which 42 patients were diagnosed with ID and 36 patients received iron supplementation (A^−^,ID^+^,T^+^). 791 patients were anaemic (36.1%), of which 276 patients were diagnosed with IDA and 215 patients received iron supplementation (A^+^,ID^+^,T^+^) (Fig. 1). The prevalence of ID was 63% in patients with Hb levels < 8 g/dl, 47.2% in patients with Hb from 8.0 to 8.9 g/dl, and 44.3% in patients with Hb from 9 to 9.9 g/dl (Fig. 2). Of the 791 anaemic patients, 64% suffered from mild, 30.2% from moderate, and 5.8% from severe anaemia. Age and body mass index (BMI) were similar across the 4 groups, while differences were observed in sex distribution and American Society of Anaesthesiologists (ASA) score. Preoperative Hb levels were 14.2 (± 1.1) g/dl in group A^−^; 13.2 (± 0.9) g/dl in group A^−^,ID^+^,T^+^; 11.1 (± 1.3) g/dl in group A^+^; and 10.5 (± 1.5) in group A^+^,ID^+^,T^+^ (Table 1).

**Table 1:** Demographic data

		A^−^ (*n* = 1,364)	A^−^, ID^+^, T^+^ (*n* = 36)	A^+^ (*n* = 576)	A^+^, ID^+^, T^+^ (*n* = 215)
Age		73.6 (± 5.8)	74 (± 5.4)	75.1 (± 6.2)	76.3 (± 6.3)
Gender (female)		28.4% (*n* = 388)	54.1% (*n* = 20)	25.5% (*n* = 150)	42.6% (*n* = 95)
BMI		27.1 (± 4.8)	24.6 (± 4.7)	26.4 (± 5.1)	26.5 (± 4.6)
ASA	I-II	17.9% (*n* = 244)	21.6% (*n *= 8)	6.8% (*n* = 39)	6.9% (*n* = 15)
	III-IV	81.9% (*n* = 1,117)	78.4% (*n* = 28)	92.3% (*n* = 532)	92.2% (*n* = 198)
	V	0.2% (*n* = 3)	0% (*n* = 0)	0.9% (*n* = 5)	0.9% (*n* = 2)
**Surgical discipline**					
	Cardiac thoracic surgery	52.3% (*n* = 713)	37.8% (*n* = 13)	44.7% (*n* = 258)	36.3% (*n* = 78)
	Urology	17.8% (*n* = 243)	13.5% (*n* = 5)	8.0% (*n* = 46)	7.2% (*n* = 16)
	Vascular surgery	13.3% (*n* = 181)	13.5% (*n* = 5)	20.7% (*n* = 119)	25.1% (*n* = 54)
	Visceral surgery	11.7% (*n* = 160)	24.3% (*n* = 9)	21.9% (*n* = 126)	27.8% (*n* = 60)
	Maxillofacial surgery	4.4% (*n* = 60)	10.8% (*n* = 4)	2.0% (*n* = 12)	3.1% (*n* = 6)
	Orthopaedic/trauma surgery	0.4% (*n* = 6)	0% (*n* = 0)	2.4% (*n* = 14)	0.4% (*n* = 1)
	Gynaecology	0.1% (*n* = 1)	0% (*n* = 0)	0.2% (*n* = 1)	0% (*n* = 0)
**Preoperative Hb (g/dl)**^**a**^		14.2 (± 1.1)	13.2 (± 0.9)	11.1 (± 1.3)	10.6 (± 1.5)
		14.1(13.4; 14.9)	13.2 (12.5; 13.6)	11.3 (10.4; 12.2)	11 (9.6; 11.8)
	female	13.4 (± 1)	12.9 (± 0.8)	10.6 (± 1.1)	10.5 (± 1.4)
		13.3 (12.7; 14)	12.7 (12.3; 13.6)	10.8 (10; 11.4)	10.8 (9.6; 11.5)
	male	14.5 (± 1)	13.6 (± 0.8)	11.3 (± 1.3)	10.7 (± 1.5)
		14.4 (13.7; 15.1)	13.4 (13; 13.7)	11.5 (10.6; 12.4)	11.1 (9.6; 12)

^a^*Hb* haemoglobin, first Hb level in the course of treatment

### Intravenous iron supplementation

**Fig. 3:**
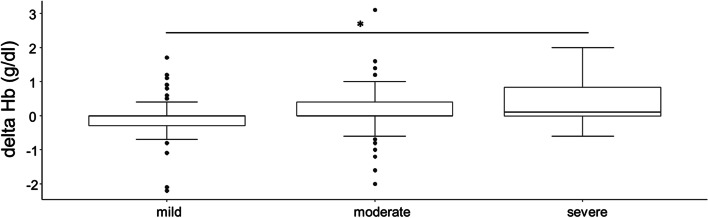
Haemoglobin (Hb) increment after iv iron depending on anaemia form (mild, moderate, severe). *Statistically significant. Mild versus severe anaemia (*p* < 0.001)

**Fig. 4:**
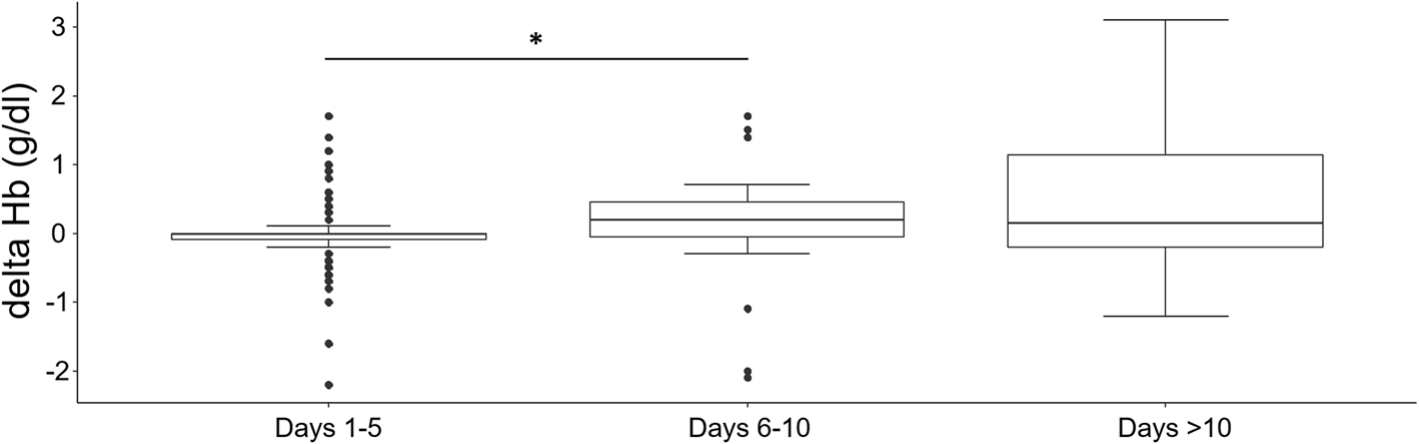
Haemoglobin (Hb) increment after iv iron depending on time of supplementation. Hb increase after iv iron supplementation 1–5 versus 6–10 days before surgery (*p < 0.001)

Time of preoperative assessment varied between surgical disciplines: 1 (1; 3) day for cardiac thoracic surgery, 1 (1; 2) day for vascular surgery, 1 (1; 3) day for orthopaedic/trauma surgery, 6 (2; 12) days for visceral surgery, 6 (3.5; 11.5) days for gynaecology, 7 (6; 10) days for urology, and 16 (5.25; 25) days for maxillofacial surgery (Supplemental Fig. 1). In group A^+^,ID^+^,T^+^ iv iron was administrated a median of 2 (1; 7) days before surgery and 42.7% received iv iron 1 day before surgery (Supplemental Fig. 2). Of those, 47.3% underwent cardiac thoracic, 33% vascular, 17.6% visceral, and 2.2% urology surgery. Iron supplementation resulted in a preoperative increase in median Hb levels of 0 (-0.3; 0) g/dl in patients with mild (*n* = 85), 0 (0; 0.4) g/dl in patients with moderate (*n* = 51), and 0.1 (0; 0.8) g/d in patients with severe (*n* = 20) anaemia (*p* < 0.001 for comparison mild vs. severe) (Fig. 3). In severely anaemic patients an Hb increase of 0.6 (0.4; 1.2) g/dl and 1.2 (0.7; 1.6) g/dl was detected with iron supplementation 6–10 and > 10 days before surgery, respectively (Supplemental Table 1). Irrespective of anaemia severity an Hb increase of 0 (-0.1; 0) g/dl was observed in patients with iron supplementation 1–5 days before surgery, 0.2 (-0.1; 0.5) g/dl in patients supplemented 6–10 days before surgery and 0.2 (-0.2; 1.1) g/dl in patients supplemented > 10 days before surgery (*p* < 0.001 for comparison 1–5 vs. 6–10 days) (Fig. 4). In iv iron supplemented A^+^,ID^+^,T^+^ patients 58% showed an increase in Hb > 0.5 g/dl (Supplemental Table 2, Supplemental Fig. 3). In total, 703 patients underwent cancer associated surgery, of which 341 interventions were visceral surgeries. In these patients a preoperative increase of median Hb levels in 0 (-0.4; 0.3) g/dl and 0.4 (0.1; 0.5) g/dl was observed after iron supplementation 1–5 and > 6 days before surgery, respectively (*p* = 0.002).

Of the A^+^ patients, 61 had ID (A^+^, ID^+^, T^−^). The majority of the patients were screened 0–1 day before surgery (*n* = 25) and supplementation of iv iron was not possible because of organisational reasons. Relative contraindications for iv iron substitution were present in 19 patients, including elevated CRP level (*n* = 13), liver or other diseases (*n* = 3), and intake of antibiotics (*n* = 1). In patients screened ≥ 2 days before surgery we found a Hb decrease by 0.3 ± 0.8 g/dl.

Using a linear regression analysis with fixed effects, we found that iron supplementation (*p* = 0.039) and severe anaemia (*p* = 0.01) were predictors for Hb increase but not gender, age, and BMI (Supplemental Fig. 4).

A preoperative Hb decrease was observed in 11% of group A^−^, 58% of group A^−^,ID^+^,T^+^, 43% of group A^+^, and 51% of group A^+^ID^+^T^+^. In group A^−^, Hb declined by ≤ 0.5 g/dl in 45% of the patients, -0.6 to -1 g/dl in 26% of patients, and − 1.6 to -2 g/dl in 17% of patients. In group A^+^ an Hb decrease by ≤ 0.5 g/dl was observed in 54% of patients, -0.6 to -1 g/dl in 20% of patients, and − 1.1 to -1.5 g/dl in 17% of patients. In patients of group A^+^ID^+^T^+^ a Hb decrease by ≤ 0.5 g/dl was observed in 55%, -0.6 to -1 g/dl in 30%, -1.1 to -1.5 g/dl in 7% (Supplemental Fig. 5). Using linear regression analysis with fixed effects, we found that age (*p* < 0.001), preoperative Hb value (*p* < 0.001) and CRP level (*p* = 0.02) were significant predictors for an Hb decrease but not gender, ferritin level, iron supplementation, or ASA score (Supplemental Fig. 6). No adverse events were observed after iv iron supplementation.

### Transfusion

**Fig. 5:**
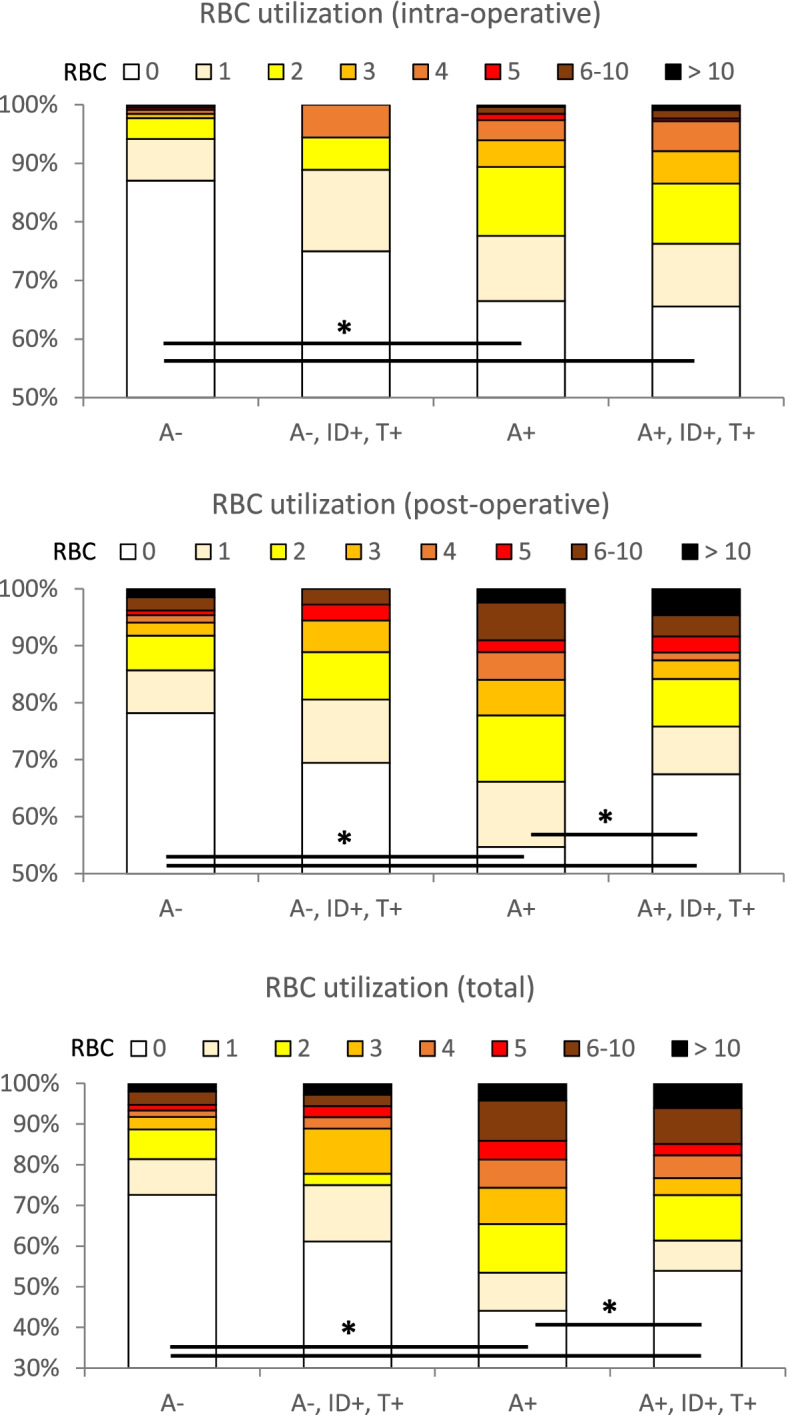
Perioperative utilization of red blood cell (RBC) units. A^−^ (no anaemia); A^−^,ID^+^,T^+^ (no anaemia, iron-deficient, iron supplementation); A^+^ (anaemia); and A^+^,ID^+^,T^+^ (anaemia, iron-deficient, iron supplementation). RBC utilization intraoperative A^−^ versus A^+^ and A^−^ versus A^+^,ID^+^,T^+^ (*p < 0.001); RBC utilization postoperative A^−^ versus A^+^, A^−^ versus A^+^,ID^+^,T^+^, and A^+^,ID^+^,T^−^ versus A^+^,ID^+^,T^+^, (*p < 0.001); total RBC utilization A^−^ versus A^+^, A^−^ versus A^+^,ID^+^,T^+^, and A^+^,ID^+^,T^−^ versus A^+^,ID^+^,T^+^, (*p < 0.001)

The number of transfused patients was consistently higher in anaemic compared to non-anaemic patients. Overall, 27% of A^−^ patients, 39% of A^−^,ID^+^,T^+^ patients, 56% of A^+^ patients, and 46% of A^+^,ID^+^,T^+^ patients required RBC transfusion (Fig. 5). During the postoperative period, A^+^,ID^+^,T^+^ patients required fewer RBC transfusions than A^+^ patients (45% in A^+^ vs. 33% in A^+^,ID^+^,T^+^). The total number of transfused RBC units was significantly lower in A^+^,ID^+^,T^+^ patients supplemented with iron (0 (0; 3)) compared to non-treated anaemic patients (1 (0; 4)) (*p* = 0.03) (Table 2). In group A^+^, 61 patients had ID and received no iv iron supplementation before surgery (A^+^,ID^+^,T^−^). The subgroup analysis showed, that the transfusion rate was higher in A^+^,ID^+^,T^−^ patients compared to A^+^,ID^+^,T^+^ patients (Supplemental Fig. 7). The number of transfused patients was significantly reduced in group A^+^,ID^+^,T^+^ with iv iron supplementation 6 days before surgery (54% in A^+^,ID^+^,T^−^ vs. 33% in A^+^,ID^+^,T^+^; *p* = 0.01) (Supplemental Fig. 7B). In the postoperative period, A^+^,ID^+^,T^+^ patients with iv iron supplementation 6 days before surgery required significantly fewer RBC transfusions compared with A^+^,ID^+^,T^−^ patients (37% in A^+^,ID^+^,T^−^ vs. 20% in A^+^,ID^+^,T^+^; *p* = 0.04). The total number of transfused RBC units was significantly lower in A^+^,ID^+^,T^+^ patients supplemented with iv iron 6 days before surgery (0 (0; 2)) compared to A^+^,ID^+^,T^−^ patients (1 (0; 2)) (*p* = 0.01).

**Table 2:** Number of transfused red blood cell units

	A^−^	A^−^ID^+^T^+^	A^+^	A^+^ID^+^T^+^
Intra	0 (0; 0.3)	0 (0; 0.3)	0 (0; 1)	0 (0; 1)
Post	0 (0; 0)	0 (0; 1)	0 (0; 2)	0 (0; 1)
Total	0 (0; 1)	0 (0; 1.25)	1 (0; 4)	0 (0; 3)

The total number of transfused red blood cell units was significant lower in A^+^ID^+^T^+^ compared to A^+^ (*p* = 0.03). A^−^ (no anemia); A^−^,ID^+^,T^+^ (no anemia, iron-deficient, iron supplementation); A^+^ (anemia); and A^+^,ID^+^,T^+^ (anemia, iron-deficient, iron supplementation)

### Hospital stay, mortality and re-operation

The re-operation rate was higher in patients with anaemia (18.8% in A^+^) compared to non-anaemic patients (10.8% in A^−^) (*p* < 0.001), or anaemic patients with iron supplementation (14% in A^+^ID^+^T^+^) (*p* = 0.14). Patients with anaemia (10.1% in A^+^) showed a higher mortality rate compared to non-anaemic patients (5.1% in A^−^) (*p* < 0.001), or patients with iron supplementation (8.3% in A^−^,ID^+^,T^+^ (*p* = 1) and 8.4% in A^+^ID^+^T^+^ (*p* = 0.36)). LOS was significantly longer (*p* < 0.001) in patients with anaemia (10 (7; 17) days in A^+^) and anaemic patients with iron supplementation (11 (7; 17) days in A^+^ID^+^T^+^) compared to non-anaemic patients (8 (7; 11) days in A^−^). No significant difference in LOS was found between group A^+^ID^+^T^+^ (11 (7; 17) days) and group A^+^ (10 (7; 17) days) (Supplemental Fig. 8). Using a linear regression analysis with fixed effects, we found that CRP level (*p* < 0.001), transfused RBC units (*p* < 0.001) and moderate anaemia (*p* = 0.04) were predictors for prolonged LOS but not gender, BMI, ID, iron supplementation, preoperative Hb increase, mild or severe anaemia (Supplemental Fig. 9). Mean Hb levels at discharge were 10.6 (± 1.5) g/dl in group A^−^, 10.5 (± 1.4) g/dl in group A^−^,ID^+^,T^+^, 9.4 (± 1.2 g/dl) in group A^+^, and 9.6 (± 1) g/dl in group A^+^ID^+^T^+^.

### Mobility

The timespan to achieve mobility was the shortest in group A^−^ (4 (2; 6) days), followed by A^−^,ID^+^,T^+^ (4.5 (3; 6) days), A^+^ (5 (3; 7) days) and A^+^,ID^+^,T^+^ (6 (3; 8) days). Anaemic patients with iron supplementation > 6 days before surgery achieved mobility 2 days earlier than patients with iron supplementation < 6 days (4 (2.8; 8.3) vs. 6 (4; 7) days). In addition, A^+^,ID^+^,T^+^ patients with iron supplementation > 6 days before surgery achieved mobility 1 day earlier than A^+^,ID^+^,T^−^ patients (4 (2; 8) vs. 5 (3; 6) days). Using linear regression analysis with fixed effects we found that LOS (*p* < 0.001) and postoperative administration of RBC units (*p* < 0.001) were predictors for delayed mobility but not gender, age, preoperative Hb level, severity of anaemia, CRP level, ASA, preoperative Hb increase, and intraoperative administration of RBC units (Supplemental Fig. 10).

## Discussion

The worlds’ elderly population is growing and consequently, the majority of surgical patients will consist of elderly patients [26]. About 1/3 of surgical patients suffer from preoperative anaemia and thus face an increased risk developing postoperative complications [5]. Therefore, it is important that anaemia, particularly in elderly patients, receives adequate attention in clinical practice and is not considered simply a normal component of aging. Out of 4,381 major surgical patients, 2,381 (54.3%) were older than 65 years with an ID prevalence of up to 63% confirming the urgent need for comprehensive anaemia management. Iron supplementation > 6 days before surgery was associated with a mild Hb increase of 0.2 g/dl. Similar results were shown in our previous work, in which we found an Hb increase of 0.2 (0; 0.7) g/dl after 6 days of iron supplementation in patients ≥ 18 years of age [21]. Interestingly, we observed a preoperative Hb decrease of more than 0.6 g/dl in 55% of group A^−^, 75% of group A^−^,ID^+^,T^+^, 46% of group A^+^, and 45% in A^−^,ID^+^T^+^, which might be associated with a progressive decrement in bone marrow haematopoiesis, neo-adjuvant therapy, advancing age, or continuous blood loss, for example in carcinomas. However, this effect can be mitigated by supporting erythropoiesis with iron supplementation [13]. Thus, the benefit of iron supplementation therapy in this patient group may not be defined by an increase in the Hb value, but by the absence of an Hb decrease. Approximately one-third of all anaemia cases in elderly patients are attributed to renal disease and chronic inflammation. The expression of hepcidin, a key regulator in iron metabolism, is elevated in patients with systemic inflammation or infection thereby reducing the supply of erythropoietic iron. In addition, patients with chronic inflammation, often suffer from impaired renal production of erythropoietin, therefore the treatment of anaemia of inflammation with erythropoietin and iv iron might be beneficial and should be considered on an individual basis. Elderly patients tend to have elevated levels of pro-inflammatory markers [27–29] which could explain the Hb decrease in some of the analysed patients. Systemic immune activation is associated with altered iron metabolism through upregulation of hepcidin. In fact, our linear regression model revealed that age and elevated CRP level were significant predictors for a Hb decrease. Based on our study design we were not able to distinguish anaemia of chronic inflammation or kidney disease from ID which could explain the observed Hb decrease or minor Hb increase. It is noteworthy, that we investigated the effect of iv iron supplementation in a reasonably heterogeneous group. For instance, patients with a gastro-intestinal malignancy undergoing preoperative chemotherapy with potential bone marrow suppression may respond differently to iron supplementation compared to patient without malignancy. Here, we showed that iron supplementation in patients undergoing cancer-related surgery was associated with a Hb increase of 0.4 g/dl before surgery.

Non-anaemic patients achieved mobility after surgery 1 day earlier compared to anaemic patients. We did not detect significant differences between non-treated and treated anaemic patients, though mobility was achieved 2 days earlier when iron was administrated > 6 days before surgery. It is worth to mention, that physiotherapy was carried out irregularly and not on weekends, and the therapists may not have been consistent in daily assessments leading to an underestimation of the time of mobility.

Overall, our analysis revealed that iron supplementation may not be associated with reduced LOS in elderly patients. One reason for this observation could be the multi-morbidity of elderly patients. Although iron supplementation may have positive effects, LOS is substantially influenced by factors including wound healing, comorbidities such as renal impairment [30], diabetes or COPD [31], postoperative complication rate [32], and frailty [33] which were not addressed in our study. To reveal a strong association between iron supplementation and LOS future studies should consider additional endpoints, particularly in studies including elderly patients. In addition, the age cut-offs for considering patients to be “elderly” is controversial [34], and results may differ for different age ranges. We considered ≥ 65 years as elderly [35–38] and therefore our observations may not apply to patients ≥ 80 years of age.

The anaemia walk-in clinic was established at the University Hospital Frankfurt in 2014 and the workflow to detect and treat ID, irrespective of the presence of anaemia, has constantly evolved with the aim to use the maximum time span for iron supplementation before surgery. However, 42.7% of the patients ≥ 65 years old received iron supplementation only 1 day before surgery suggesting that surgery in these patients was urgent. In addition, we cannot exclude the possibility that appointments were planned and scheduled on short notice. The fact that a large proportion of patients received iron shortly before surgery illuminates a clear need for action. Although the patients were correctly identified, there is still a need for optimization. The majority of these patients underwent cardiac thoracic or vascular surgery and were invited to the hospital for preoperative assessment 1 day before surgery. To optimize our screening process, we will invite these patients to the anaemia walk-in clinic separately and independent of their appointment for preoperative assessment in future. Spahn et al. analysed the effects of ultra-short-term administration of iv iron/erythropoietin/vitamin B12/folic acid 1 day before surgery in cardiac patients. The combination significantly reduced the number of RBC transfusions from a median of 1 (0; 3) unit in the patient group without treatment to 0 (0; 2) units in the treatment group during the first 7 days after surgery (OR 0.7, 95% CI 0.50–0.98; *P* = 0.036). In addition, patients with treatment had higher Hb concentrations during the first 7 days after surgery compared to patients without treatment (*P* < 0.001) [39]. Overall, the increasing body of evidence suggests that iron supplementation should be provided any time to replenish iron stores before surgery. In addition, the cause of anaemia is multifactorial, particularly in elderly surgical patients. However, anaemia caused by malnutrition or malabsorption of nutrients, such as vitamin B12 and folate, can be easily treated with oral or iv supplements. To provide a comprehensive anaemia management we are in the process of extending our anaemia management algorithm to include diagnosis and treatment for folate and/or vitamin B12 deficiencies.

The association between delaying surgery to stimulate erythropoiesis in anaemic patients and perioperative outcome is still a questionable issue and controversial results have been published. Forni et al., revealed that surgical delay was not associated with a higher mortality rate. In fact, after analysis of almost 24,000 elderly patients with hip fracture, the authors reported a significantly lower mortality rate in patients with a multidisciplinary treatment approach compared to patients in the usual care model [11]. In line with this observation Librero et al. showed that among others advancing age, male gender, and chronic comorbidity were associated with higher mortality, but the timing of surgery was not [40]. In addition, Hangaard Hansen et al. conducted a systematic review including > 13,500 patients and found no association between treatment delay and survival rate in colon cancer patients [41]. In contrast, several studies demonstrated that early surgery can reduce mortality rate in patients with hip fracture [42–46]. Since the number of surgical elderly patients is increasing, the need for a multidisciplinary approach is inevitable.

Finally, our analysis revealed that iron supplemented patients required fewer RBC transfusions compared to non-treated anaemic or non-treated IDA patients, supporting the beneficial effect of iron in these patients. It is noteworthy, that anaemia in women was still considered as < 12 g/dl and not < 13 g/dl in our hospital. The classification of anaemia for non-pregnant women postulated by the WHO has been the focus of many discussions in the past years [22]. Therefore, accepting a Hb level of 12 g/dl or less for women in the pre-surgery assessment while mean have a higher threshold of 13 g/dl, makes transfusion, with all its associated side effects and complications, more likely in women. In addition, the lower Hb threshold could preclude a large proportion of women undergoing surgery from receiving effective treatment [47].

## Conclusions

To the best of our knowledge, this is the first study revealing the prevalence of ID in elderly surgical patients. Our results indicate that iron supplementation in elderly surgical patients with IDA was associated with an Hb increase of up to 1.2 g/dl, which is equivalent to the transfusion of 1 to 1.5 allogenic RBC units. Accordingly, iron supplementation was associated with a significantly reduced transfusion rate. Thus, iron supplementation is an effective therapeutic agent to support preoperative management and to reduce the use of the increasingly diminishing resource of allogeneic blood. Particularly in patients with continuous blood loss before surgery, counteracting a decrease in Hb values before surgery is a significant benefit. Moreover, mobility was increased in the elderly patients who received treatment > 6 days before surgery. Interestingly, we found that a large proportion of the elderly patients were scheduled for surgery on short notice, highlighting the need for comprehensive anaemia management before hospital admission. In addition, postponing surgery in this vulnerable group of patients should be considered to optimize the treatment of iron deficiency anaemia. Geriatricians may be able to identify preoperative anaemia and act to reduce its associated risks, thus improving outcomes for the steadily increasing number of elderly patients undergoing major surgery.

## Supplementary Information

**Additional file 1.**

## Data Availability

The datasets used and/or analysed during the current study are available from the corresponding author on reasonable request. The manuscript has not been and will not be submitted, in part or entirely, elsewhere for publication and if accepted, the paper will not be published elsewhere.

## Abbreviations

A+: Anaemia
A-: No anaemia
A+,ID+,T+: Anaemia, ID and iron supplementation
A-,ID+,T+: No anaemia, ID, and iron supplementation
ASA: American Society of Anaesthesiologists
ID: Iron deficient
IDA: Iron deficiency anaemia
IQR: Interquartile ranges
iv: Intravenous
LOS: Length of stay
PBM: Patient Blood Management
RBC: Red blood cell
SD: Standard deviation
WHO: World Health Organization
