# Supplementary material for: The impact of intravenous iron supplementation in elderly patients undergoing major surgery
Source: BMC Geriatr. 2022 Apr 7;22:293. doi: 10.1186/s12877-022-02983-y (PMC8988356; doi:10.1186/s12877-022-02983-y)
Supplement: Supplementary file 1 — Additional file 1. [file 12877_2022_2983_MOESM1_ESM.docx]

# Supplemental_Figure_1

Time for preoperative assessment for screening iron deficiency before surgery

# Supplemental_Figure_2


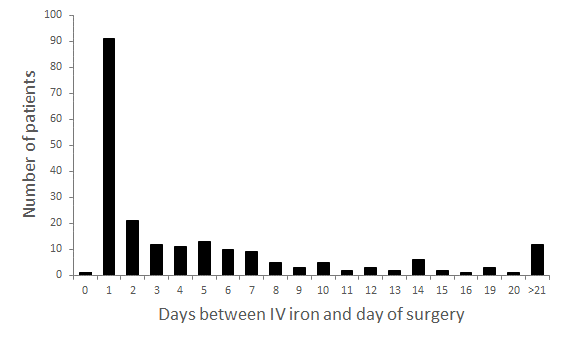


Number of iron deficient anaemic patients receiving iron supplementation before surgery. 91 patients received intravenous iron supplementation 1 days before surgery. Of these, 43 patients underwent cardiac thoracic surgery, 30 vascular surgery, 16 visceral surgery, and 2 urology

# Supplemental_Figure_3


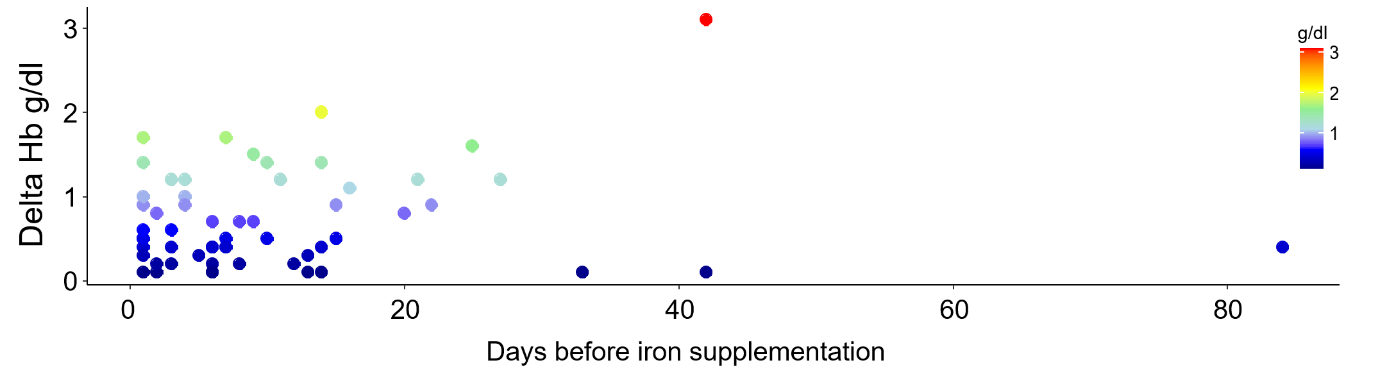


Delta hemoglobin (Hb) g/dl depended on time of iron supplementation before surgery

# Supplemental_Figure_4


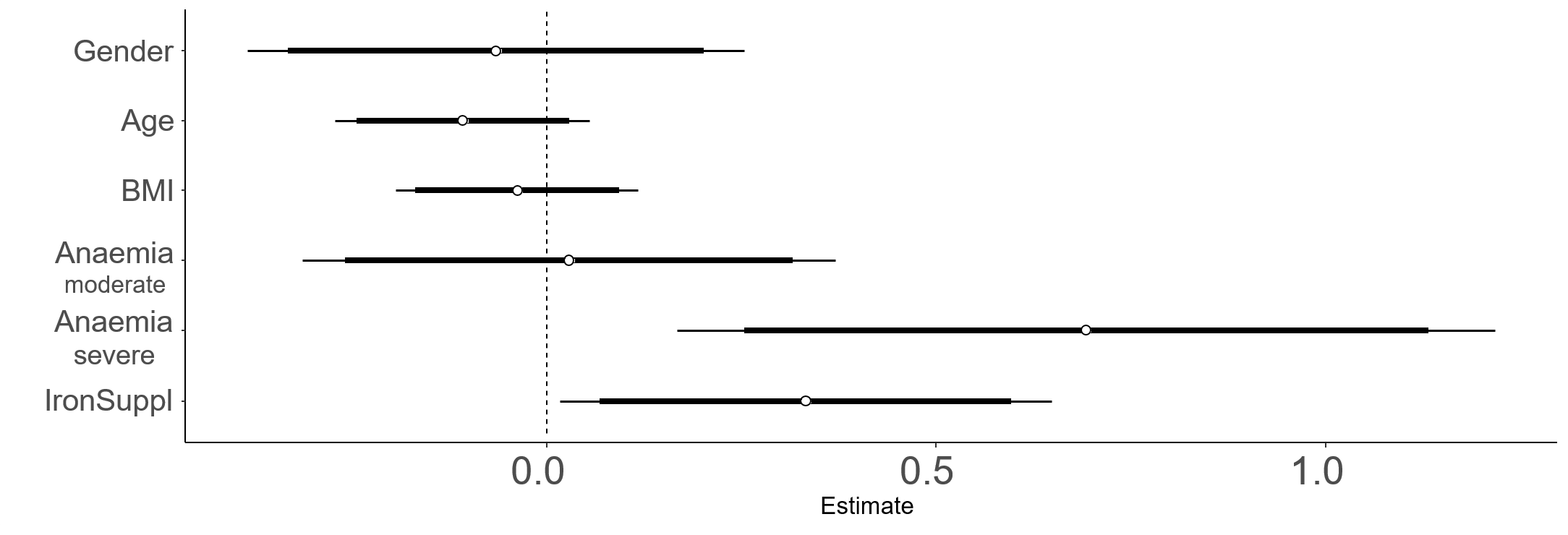


Results of linear regression analysis to assess predictors for haemoglobin increase

# Supplemental_Figure_5

Preoperative hemoglobin (Hb) g/dl decrease. A^-^ (no anemia); A^-^,ID^+^,T^+^ (no anemia, iron-deficient, iron supplementation); A^+^ (anemia); and A^+^,ID^+^,T^+^ (anemia, iron-deficient, iron supplementation)

Supplemental_Figure_6


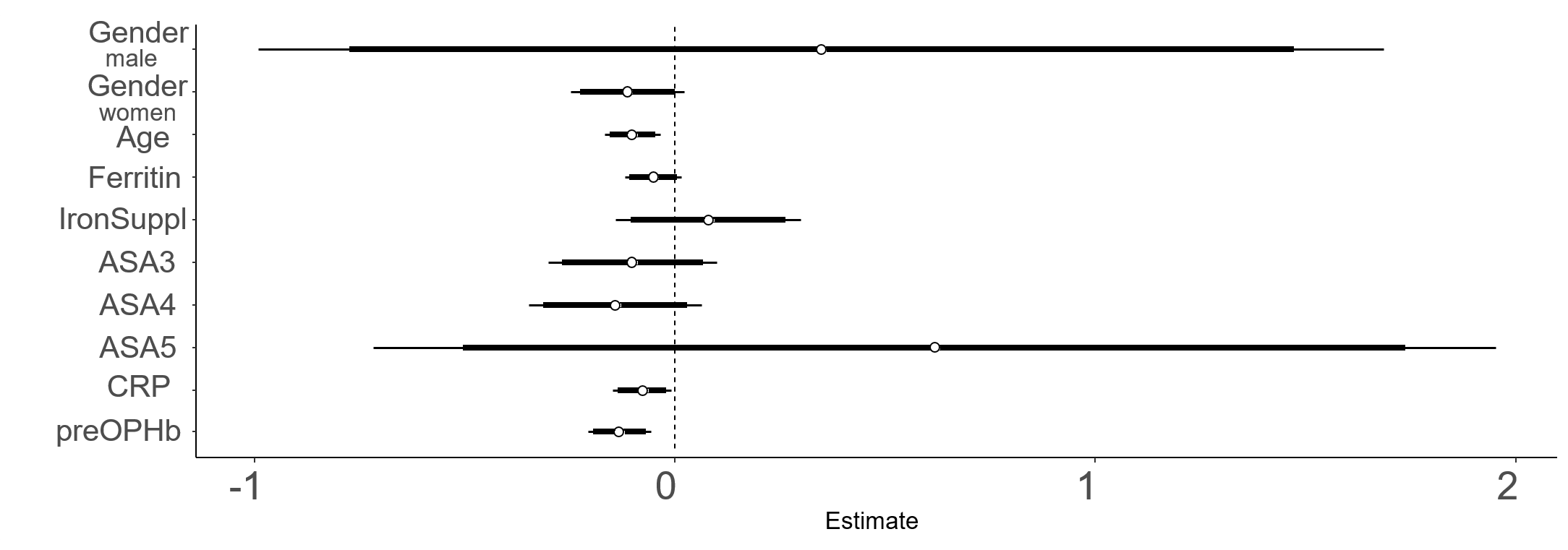


Results of linear regression analysis to assess predictors for haemoglobin decrease

Supplemental_Figure_7


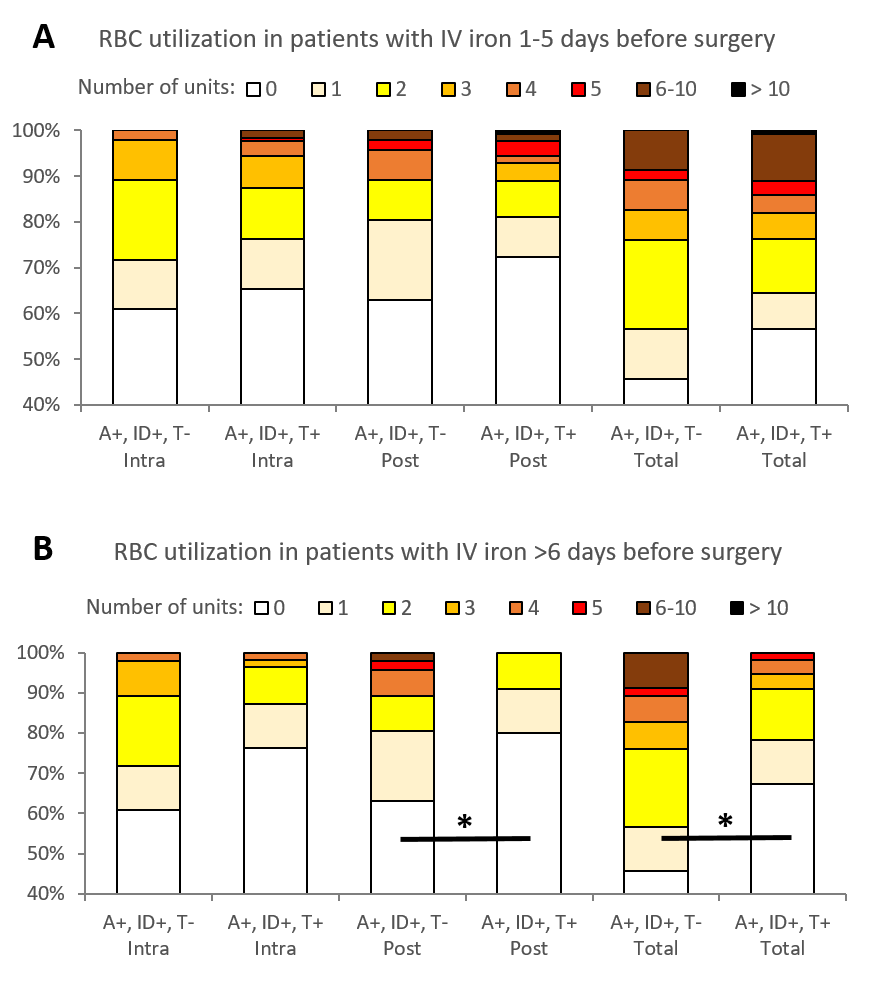


Perioperative utilization of red blood cell (RBC) units. A^+^,ID^+^,T^-^ (anaemia, iron-deficient, NO iron supplementation), A^+^,ID^+^,T^+^ (anaemia, iron-deficient, iron supplementation 6 days before surgery). Intra=intraoperative, Post=postoperative. *Statistically significant. RBC utilization postoperative A^+^,ID^+^,T^-^ versus A^+^,ID^+^,T^+^ (p=0.04) and intraoperative A^+^,ID^+^,T^-^ versus A^+^,ID^+^,T^+^ (p=0.01)

# Supplemental_Figure_8


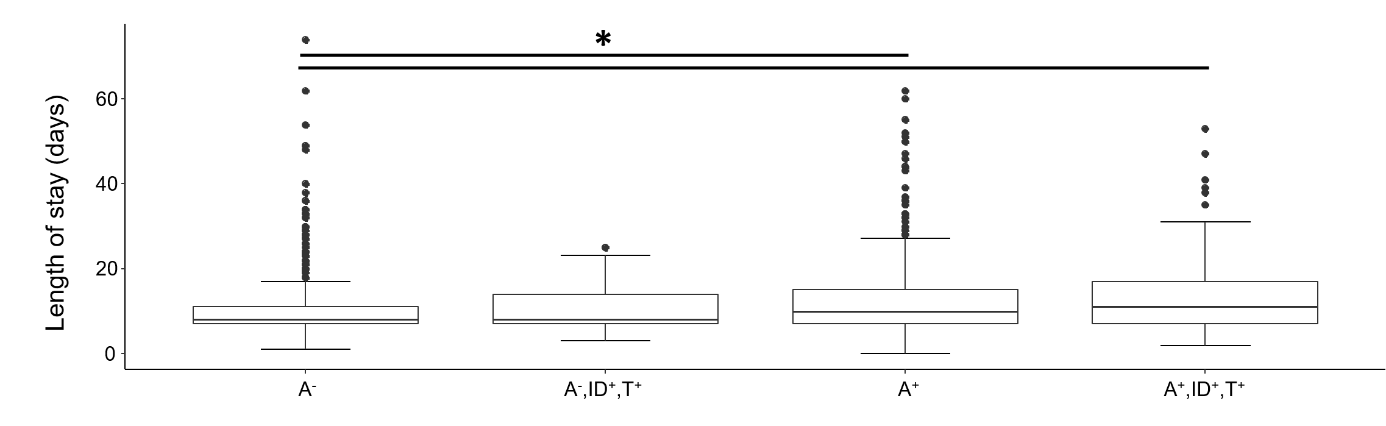


Hospital length of stay. *Statistic significant. A^-^ versus A^+^ (p<0.001) and A^-^ versus A^+^,ID^+^,T^+^ (p<0.001)

Supplemental_Figure_9


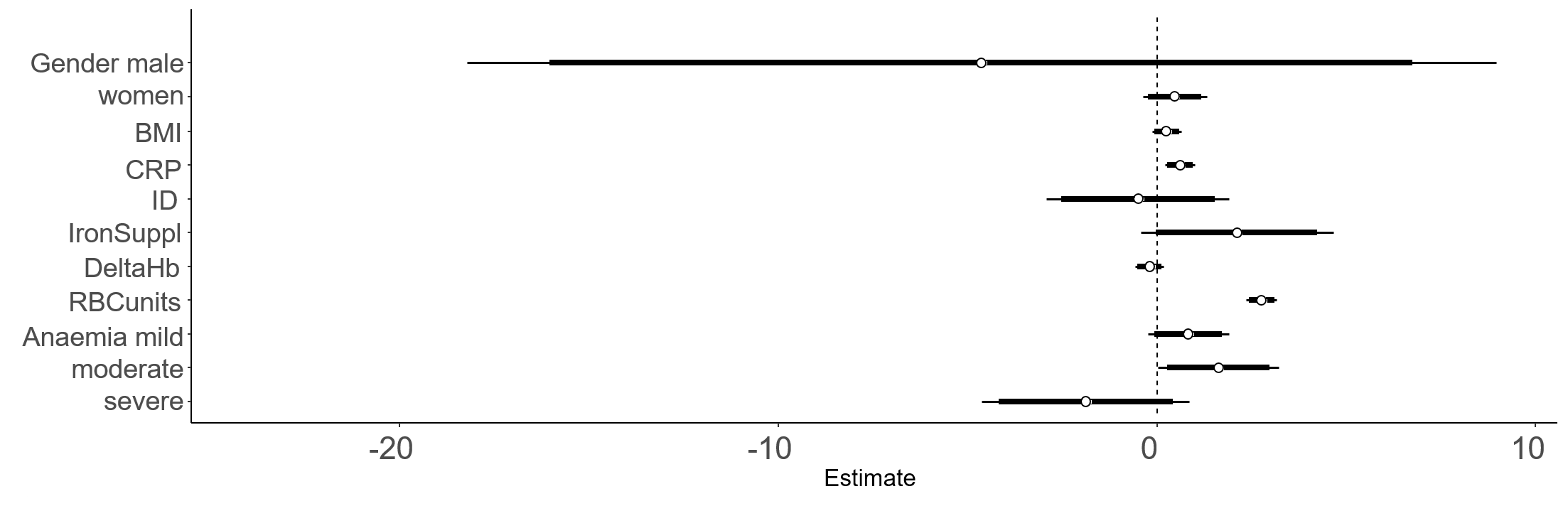


Results of linear regression analysis to assess predictors for prolonged hospital stay.

Supplemental_Figure_10


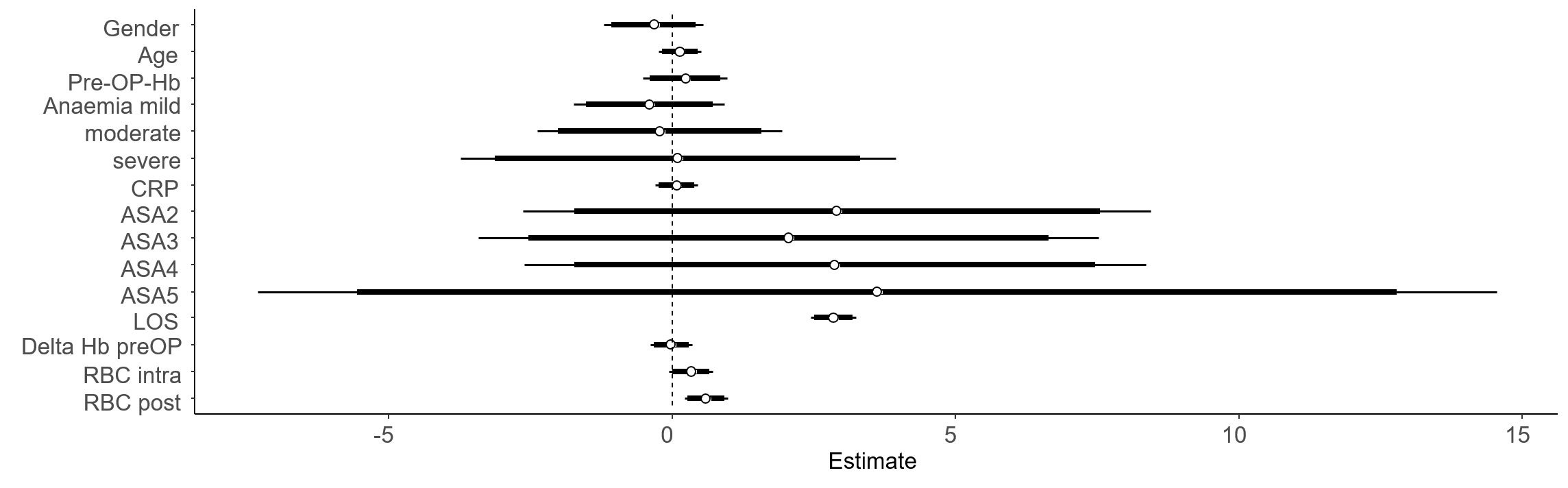


Results of linear regression analysis to assess predictors for delayed mobility.

# Supplemental_Table_1 Hemoglobin increase depending on time of iron supplementation

|  | **days 1-5**  **(g/dl)** | **days 6-10**  **(g/dl)** | **days >10**  **(g/dl)** |
| --- | --- | --- | --- |
| mild | 0 (-0.2; 0.0) | 0.1 (-0.3; 0.4) | -0.1 (-0.3; 0.9) |
| moderate | 0 (0.0; 0.0) | 0.1 (-0.1; 0.3) | 0.2 (-0.1; 1) |
| severe | 0 (-0.1; 0.1) | 0.6 (0.4; 1.2) | 1.2 (0.7; 1.6) |

# Supplemental_Table_2 Amount of iron deficient anaemic responders depending on time of iron supplementation

|  | **Days 1-5** | **Days 6-10** | **Days >10** | **All days** |
| --- | --- | --- | --- | --- |
| 0.1-0.5 g/dl | 38% | 53% | 40% | 42% |
| >0.5 g/dl | 62% | 47% | 60% | 58% |
